# Integrating human papillomavirus (HPV) vaccine into China's National Immunization Program: global lessons and strategic considerations

**DOI:** 10.1016/j.pmedr.2026.103590

**Published:** 2026-07-27

**Authors:** Danjie Jiang, Yan Zhang, Dandan Zhang

**Affiliations:** Ningbo Municipal Center for Disease Control and Prevention, Ningbo, China

**Keywords:** Human papillomavirus vaccine, National Immunization Program, China, School-based delivery, Vaccine hesitancy

## Abstract

**Objective:**

To review global evidence on human papillomavirus (HPV) vaccine introduction and delivery strategies, and derive actionable lessons for China's ongoing national rollout following its integration of HPV vaccine into the national immunization program in November 2025.

**Methods:**

This is an analytical essay that synthesises published evidence on HPV vaccination programs worldwide, with a focus on delivery strategies, coverage outcomes, and implementation barriers. Literature was identified through PubMed and WHO databases for the period 2012–2026. No primary data collection was conducted.

**Results:**

As of June 2020, 107 of 194 WHO member states (55%) had introduced HPV vaccination, yet only 15% of girls worldwide completing the series. School-based delivery with grade-based targeting achieves highest coverage (e.g., Rwanda >90%), and mixed models (school-based with facility-based catch-up) represent best practice. Key barriers include vaccine hesitancy, logistical complexity, weak data systems, and policy design. In China, enablers include domestic vaccine production capacity, centralized governance, the nine-year compulsory education system, and established school-based health programs; major challenges include parental hesitancy, regional disparities, and social media misinformation.

**Conclusions:**

Drawing on global experience, China should leverage its existing health and education infrastructure to implement the current two-dose schedule effectively, strengthen multi-level communication strategies, and proactively manage vaccine confidence to achieve high and equitable coverage, contributing to global cervical cancer elimination goals.

## Highlights

• China's 2025 human papillomavirus (HPV) vaccine integration is a historic public health milestone.
• School-based delivery with age-based targeting is the recommended primary strategy.
• Sustained communication and data systems are crucial to overcome hesitancy.

## 1 Introduction

Cervical cancer poses a preventable burden, with human papillomavirus (HPV) vaccination as the cornerstone of primary prevention. In 2020, WHO launched the global strategy to eliminate cervical cancer, setting a core target of 90% HPV vaccination coverage among girls by age 15 by 2030 (World Health Organization, 2019b).

On November 10, 2025, China took a historic step by officially integrating the HPV vaccine into its national immunization program. Under this policy, girls born on or after November 10, 2011 who have reached 13 years of age are eligible to receive free two-dose bivalent HPV vaccination (administered 6 months apart). The program provides domestic bivalent HPV vaccines targeting HPV types 16 and 18. This move positions China among the growing number of nations committed to cervical cancer elimination.

However, translating policy intent into population-level impact requires navigating complex implementation challenges. While China possesses significant enabling factors—including domestic vaccine manufacturing capacity, centralized governance, a near-universal compulsory education system, and established precedents for school-based health interventions—substantial barriers remain, including pronounced parental vaccine hesitancy, large regional disparities in healthcare access, and the rapid spread of misinformation through social media platforms. This study reviews global evidence on HPV vaccine delivery strategies and barriers, with the aim of informing China's rollout by extracting lessons applicable to its unique context.

## 2 Global landscape: progress, gaps, and lessons

As of June 2020, 107 of 194 WHO member states (55%) had introduced HPV vaccination. Introduction remains highly unequal: 85% of countries in the Americas and 77% in Europe have done so, compared to only 41% of low- and middle-income countries (Bruni et al., 2021). Critically, many populous nations including India and Nigeria, had not achieved nationwide introduction as of 2020, severely constraining global coverage.

Coverage estimates for 2019 reveal the magnitude of the challenge. Worldwide, only 15% of girls completed the full HPV vaccine series. Among countries with programs, average final-dose coverage was 53%, indicating substantial dropout and vaccine hesitancy. Only 6% of introducing countries achieved the 90% target, while 40% had coverage at or below 50%. Regional disparities are stark: Australia and New Zealand reached 77% coverage, Latin America and the Caribbean 61%, while East and Southeast Asia managed only approximately 4% (Bruni et al., 2021).

These data carry three implications for China. First, delays in populous countries disproportionately hinder global progress toward the 90% coverage target. Second, vaccine introduction alone does not guarantee high coverage and program performance matters equally. Third, East Asia's low coverage underscores the need for implementation strategies that are carefully adapted to regional contexts rather than directly transplanted from Western settings.

## 3 Delivery strategies: what works

School-based delivery serves as the cornerstone of effective HPV vaccination programmes. Evidence consistently demonstrates that school-based vaccination achieves higher coverage than facility-based approaches alone. A systematic review (Felsher et al., 2024) confirmed that school-based strategies consistently outperformed other delivery models across diverse settings. In the seven-country Gardasil Access Program, school-based and mixed school-facility strategies attained approximately 93% coverage, versus 77% for facility-only delivery (Paul and Fabio, 2014). Rwanda's school-based program, combined with community mobilization, achieved over 90% coverage (Binagwaho et al., 2012).

For China, school-based delivery is particularly relevant. The country's nine-year compulsory education system provides near-universal access to the target age group, and existing school-located health programs—such as Measles-Mumps-Rubella booster shot vaccination, and tuberculosis screening—offer operational precedents and established coordination mechanisms between health and education authorities at various administrative levels.

Age-based eligibility criteria also play a critical role in programme success. A cluster-randomized trial in Tanzania found that using grade rather than age as eligibility criteria yielded significantly higher coverage (78.7% vs. 72.1%) (Watson-Jones et al., 2012). Grade provides administrative simplicity and precision in student identification, a finding particularly relevant for China's school-based rollout. China's current policy specifies age 13 as the eligibility criterion—a decision that balances epidemiological considerations (vaccinating before the onset of sexual activity, when immune response is optimal) with programmatic feasibility.

Health facilities serve as essential complements: even robust school programs require mechanisms to reach absentees, dropouts, and out-of-school girls. Mixed delivery models—school-based routine vaccination supplemented by facility-based catch-up—represent best practice.

## 4 Persistent implementation challenges

Vaccine confidence and social barriers represent a major implementation challenge. Vaccine hesitancy remains a core barrier. Ontario's ten-year program evaluation found parental concerns about vaccine safety, perceived newness, and stigma linking HPV to sexual activity persistently undermined uptake (Dube et al., 2021). Similar patterns have been documented in East Asian settings—Japan's suspension of proactive recommendations following safety scares led to a dramatic coverage collapse from approximately 70% to near zero, offering a stark regional cautionary tale (Tomoi et al., 2026; Simms et al., 2020). Social media misinformation amplifies these concerns. Notably, highly educated parents sometimes exhibit greater hesitancy due to exposure to diverse—including negative—information, complicating communication strategies. In China, parental hesitancy is a significant concern that requires targeted, sustained communication efforts.

System and policy barriers further complicate the rollout. School-based programs face substantial logistical burdens: obtaining and tracking parental consent forms, coordinating with school schedules, managing multiple vaccines concurrently, and ensuring proper cold-chain maintenance all require intensive inter-sectoral coordination. Resource constraints and competing priorities can constrain program reach, particularly in under-resourced regions. Accurate coverage assessment requires reliable denominators—a challenge where population mobility is high or census data are outdated. Crucially, traditional childhood immunization indicators do not predict HPV program performance, necessitating adolescent-specific monitoring systems. Some studies report implausible coverage exceeding 100%, revealing deficiencies throughout the data chain from administrative reporting to population estimation (Pak et al., 2025). The voluntary nature of HPV vaccination in many settings contributes to lower uptake compared to school-required vaccines. In Ontario, HPV vaccination coverage (60%) lagged behind the school-required meningococcal vaccine (82%) (Dube et al., 2021). This suggests that integration with existing immunization requirements could boost uptake, though mandating must be carefully balanced against concerns about parental autonomy.

## 5 Discussion and conclusions

China's initiation of free HPV vaccination for eligible girls represents a historic public health achievement. Global evidence and China-specific contextual factors suggest several strategic considerations for successful implementation.

A first strategic consideration is that schools should serve as the primary delivery platform, which requires institutionalized collaboration between health and education sectors at all administrative levels. China's compulsory education system and existing school health programs provide a strong foundation for this approach. However, school-based programs must be complemented by accessible catch-up mechanisms through community health centers for absentees and out-of-school girls.

A second priority involves designing multi-level, sustained communication strategies. Communication must address parents, students, teachers, and communities with differentiated messages. Content should consistently frame HPV vaccine as fundamentally a “cervical cancer prevention vaccine” to mitigate stigma associated with sexual transmission. Frontline personnel—vaccinators and teachers—need training to confidently address safety concerns and counter misinformation. Communication is not a pre-launch activity but a continuous process requiring adaptive strategies as confidence fluctuates. Given the significant parental hesitancy observed in China, communication efforts should prioritize building trust through authoritative, accessible, and culturally appropriate channels.

A third essential element is investment in integrated data systems. Modern platforms connecting immunization registries, school enrollment systems, and electronic health records enable cohort-based tracking and performance monitoring. Regular coverage surveys should validate administrative data and inform program adjustments. China's advancing health information infrastructure provides opportunities to build robust monitoring systems from the outset.

Fourth, proactive management of vaccine confidence is crucial. Given global patterns of hesitancy and the rapid spread of misinformation on social media, China should establish mechanisms to monitor vaccine confidence and respond to emerging concerns. Collaborating with professional societies, key opinion leaders, and educational institutions to disseminate consensus-based information can strengthen public trust.

Fifth, addressing regional disparities remains an urgent task. China's significant urban-rural and east-west disparities in healthcare resources present a major implementation challenge. Resource allocation, technical support, and communication strategies should be adapted to local contexts, with particular attention to underserved regions where health infrastructure may be less developed.

For China, launching nationwide HPV vaccination marks a milestone achievement. Global experience suggests that realizing this policy's promise requires a resilient, context-adapted implementation framework. School-based delivery with robust education-health collaboration, targeted communication strategies addressing parental hesitancy, integrated data systems, and proactive management of vaccine confidence constitute essential pillars. China's domestic vaccine production capacity, centralized governance, compulsory education system, and established school health programs are significant enabling assets, while parental hesitancy, regional disparities, and social media misinformation require explicit mitigation strategies. Future research should examine implementation determinants in China's diverse provincial context, contributing critical evidence to global elimination efforts while ensuring that Chinese girls receive the protection they deserve.

## Author Contribution

D.J. conceived the analytical framework, conducted the literature synthesis, and drafted the initial manuscript. Y.Z. contributed to data interpretation and critical revision of the manuscript for important intellectual content. D.Z. participated in the extraction of global lessons and revised the manuscript for key arguments. All authors made significant contributions to writing and editing, reviewed the final version, and approved the submission.

## CRediT authorship contribution statement

**Danjie Jiang:** Writing – original draft, Conceptualization. **Yan Zhang:** Visualization, Supervision, Conceptualization. **Dandan Zhang:** Visualization, Supervision, Conceptualization.

## Ethics

Not applicable.

## Funding

This research was supported by Zhejiang Science and Technology Plan for Disease Prevention and Control Project (No. 2026JKZ059).

## Declaration of competing interest

The authors declare that they have no known competing financial interests or personal relationships that could have appeared to influence the work reported in this paper.

## Data Availability

No data was used for the research described in the article.

## References

[bb0005] Binagwaho A., Wagner C.M., Gatera M., Karema C., Nutt C.T., Ngabo F. (2012). Achieving high coverage in Rwanda's national human papillomavirus vaccination programme. Bull. World Health Organ..

[bb0010] Bruni L., Saura-Lazaro A., Montoliu A., Brotons M., Alemany L., Diallo M.S., Afsar O.Z., LaMontagne D.S., Mosina L. (2021). HPV vaccination introduction worldwide and WHO and UNICEF estimates of national HPV immunization coverage 2010-2019. Prev. Med..

[bb0015] Dube E., Wilson S., Gagnon D., Deeks S.L., Dubey V. (2021). “it takes time to build trust”: a survey Ontario’s school-based HPV immunization program ten years post-implementation. Hum. Vaccin. Immunother..

[bb0020] Felsher M., Shumet M., Velicu C., Chen Y.T., Nowicka K., Marzec M., Skowronek G., Pieniążek I. (2024). A systematic literature review of human papillomavirus vaccination strategies in delivery systems within national and regional immunization programs. Hum. Vaccin. Immunother..

[bb0025] Pak L., Rollison J., Rabinowitz M., Faherty L.J. (2025). Human papillomavirus vaccine coverage surveys in low- and middle-income countries: current efforts and future considerations for very young adolescents. BMJ Glob. Health.

[bb0030] Paul P., Fabio A. (2014). Literature review of HPV vaccine delivery strategies: considerations for school- and non-school based immunization program. Vaccine.

[bb0035] Simms K.T., Hanley S.J.B., Smith M.A., Keane A., Canfell K. (2020). Impact of HPV vaccine hesitancy on cervical cancer in Japan: a modelling study. Lancet Public Health.

[bb0040] Tomoi H., Hanley S.J.B., Larson H.J., Tomoi H., Masuda K. (2026). Determinants of human papillomavirus vaccination decision-making in Japan: a scoping review exploring contextual, social, and adolescent-specific influences. Vaccine.

[bb0045] Watson-Jones D., Baisley K., Ponsiano R., Lemme F., Remes P., Ross D., Kapiga S., Mayaud P., de Sanjose S. (2012). Human papillomavirus vaccination in Tanzanian schoolgirls: cluster-randomized trial comparing 2 vaccine-delivery strategies. J. Infect. Dis..

[bb0050] World Health Organization (2019). Report EB146/9. Accelerating the Elimination of Cervical Cancer as a Global Public Health Problem (16 December 2019). https://apps.who.int/gb/ebwha/pdf_files/EB146/B146_9-en.pdf.

